# Field and Laboratory Wear Tests of Machine Components Used for Renovation of Dirt Roads—A Case Study

**DOI:** 10.3390/ma16186180

**Published:** 2023-09-12

**Authors:** Jarosław Selech, Wiktor Majchrzycki, Dariusz Ulbrich

**Affiliations:** 1 Institute of Mechanical Science, Vilnius Gediminas Technical University, Plytinės 25, LT-10105 Vilnius, Lithuania; jaroslaw.selech@vilniustech.lt; 2 Faculty of Civil and Transport Engineering, Poznan University of Technology, 60-965 Poznan, Poland; wiktor.majchrzycki@gmail.com

**Keywords:** wear, dirt road, renovation, heavy machinery, field test, laboratory test

## Abstract

Renovation of dirt roads requires a reliable and durable work tool. This article includes the methodology of field and bench tests as well as the results of these tests and conclusions for cutters used for dirt road renovation. The main novelty of the research presented in this article was to determine the wear mechanisms occurring during field and laboratory tests, to determine the differences in wear levels and the cost of renovation of one kilometer of dirt road. Calculations of the efficiency of replacing these working elements and the cost of operating various cutters per km are also presented. The lowest mass loss was characterized by milling cutters Ø25 mm mounted on an expansion sleeve and amounted to 130 g. The dominant wear mechanism that was observed after the renovation of dirt roads was micro-scraping and micro-bruising. For this variant, the cost per 1 km of road renovation was also the lowest and amounted to about PLN 2.

## 1. Introduction

Machine components are subject to various types of wear throughout their lifetime [1]. These components experience different forms of degradation, each of which affects their performance and durability. Typical types of wear on machinery components include abrasion [2], corrosion or tribocorrosion [3], fatigue [4] and erosion [5]. Abrasion occurs when surfaces come into contact and rub against each other, causing material loss due to friction. This type of wear is often found in parts such as gears, bearings and belts, where repeated motion causes surface degradation. Abrasion wear is also found in machine components working in the ground [6,7,8]. Due to the interaction of the abrasive mass and the elements in it (such as stones), there is a change in the shape-dimensional properties leading to damage to the component and the need to replace it with a new one [9,10].

There are several methods to reduce abrasion wear, which is the process of materials wearing down due to frictional forces and can lead to the failure of a machine component and its stoppage, which generates financial losses and additional costs related to the restoration of operating potential. One method of reducing abrasive wear is lubrication. Lubricants, such as oils or greases, can reduce friction between surfaces and minimize abrasion [11]. The lubricant forms a protective layer that separates surfaces and reduces wear [12]. Another method used to reduce wear is the use of coatings applied to the surface of the material [13]. Application of protective coatings to the surface of a material can enhance its resistance to abrasion. These coatings can be made of various materials, such as polymers [14], ceramics [15] or metal alloys [16], which provide a harder and more durable surface. Another method of reducing wear is also to modify the surface layer of parts by applying heat treatment [17]. Heat treatments, such as quenching and tempering, can increase the hardness and strength of a material [18]. Hardened materials are less susceptible to abrasion wear as they can withstand higher frictional forces [19]. One of the simplest and most common methods of reducing wear is the proper selection of materials [20]. Materials with high hardness, toughness and wear resistance can reduce abrasion wear [21]. Hardened steel or wear-resistant alloys applied where abrasion is a concern can significantly prolong the lifespan of components. In the case of heavy machinery, it is also important to properly select the shape of components exposed to abrasive wear [22]. All of the methods described above have the effect of reducing wear on machine components, but they cannot stop this process.

Another advanced method is the use of self-lubricating composite materials [23,24], which involves incorporating fibers or particles of solid lubricating graphite into the matrix alloy. This allows for increased resistance to abrasive wear (the addition of graphite can reduce friction between the surfaces of mating parts, which in turn reduces wear and the risk of mechanical damage from friction), improved corrosion resistance and better electrical conductivity. Therefore, it is important to perform both laboratory and experimental field tests to learn about wear processes and optimize methods of reducing it.

There are a number of wear testing methods that include: the pin-on-disk tests [25], abrasion tests on laboratory stands [26,27] as well as wear rate measurements in laboratory and field conditions [28]. For the latter group of studies researchers quantify the wear rate by measuring the mass loss [29] or volume loss [30] of the tested sample over a specific duration. This allows for comparisons between different materials, coatings or experimental conditions. In addition, it allows comparison of laboratory and field test results for the same samples and modeling of the wear phenomenon. Research is also carried out on the crushing technology of road materials themselves, which will also help influence the road renovation process and reduce wear and tear on machinery [31,32]. Studies of machine components working in the soil also include attempts to assess the wear and quality of chisel work [33] and wear modeling methods [34] in the soil, which influences the estimation of working time and replacement costs of such components. Chunsong Guan and co-authors [35], in order to wear out machine components working in the ground, proposed coating the surfaces to ensure longer operation without wasting time on replacement and generating additional costs.

The main goal of the research presented in this article is to determine the wear of three types of teeth used in milling machines, which are used to renovate dirt roads. This research was carried out both in laboratory conditions on a specially prepared stand and in real operating conditions of these machines. An additional objective is to determine the most favorable, in terms of tool replacement time and economy, variant of the tooth of the milling machine used for the renovation of dirt roads. The novelty brought by the realized research is the comparison of laboratory and field test results for milling machine teeth. This will allow us to learn about the phenomena occurring during the operation of the milling machine teeth and to perform accelerated laboratory tests in terms of wear resistance of these components. The next part of this article describes the methodology of laboratory and field tests, presents obtained results with a discussion and suggests directions for further research.

## 2. Materials and Methods

The tests were performed on 3 cutters from 2 types of milling machines (Valentini RAMBO milling machines 1500 and Valentini IVAN 1500, Valentini, Camposampiero PD, Italy) used for road renovation. The basic functions of the machines mainly include the creation of an optimal granulometric curve for road construction, milling of asphalt without prior grinding, milling and recycling of asphalt roads with a surface thickness of up to 14 cm (without prior grinding of asphalt) as well as mixing the soil to improve its homogeneity and the leveling the land.

**Figure 1:**
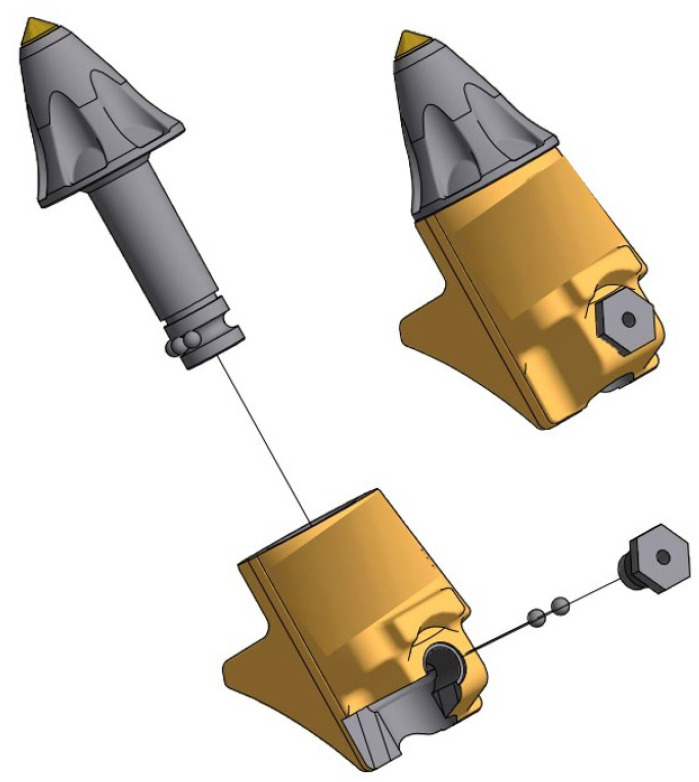
Mounting the Ø25 cutter [36].

In road renovation milling machines, the working elements can be divided into two main groups. The first is the cutter seated in slots on the rotor (Figure 1).

**Figure 2:**
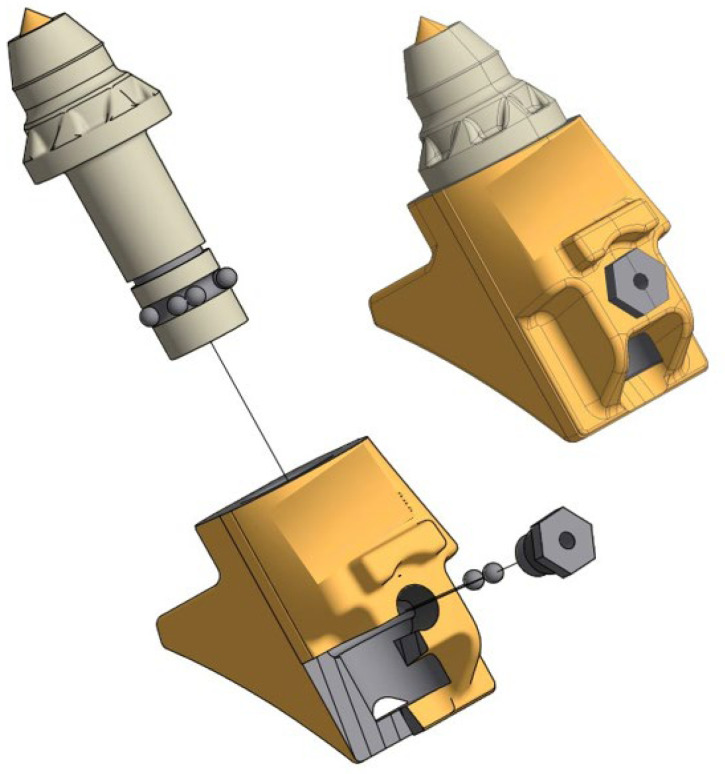
Mounting the Ø35 cutter [37].

The second group comprises the cutters and the slides as well as guards made of Hardox wear-resistant steel (Figure 2).

Due to the harsh working conditions associated with the high rotational speed required to grind road materials, as well as for the sake of the high hardness of the aggregates from which the roads are made of, the machine components wear significantly. Many types of working elements are available on the market. Three of these were selected for testing, as follows:

- Milling cutter Ø25 mm mounted on bearing balls (Ø25 mm balls);
- Milling cutter Ø25 mm mounted on expansion sleeve (Ø25 mm sleeve);
- Milling cutter Ø35 mm mounted on bearing balls (Ø35 mm balls).

**Figure 3:**
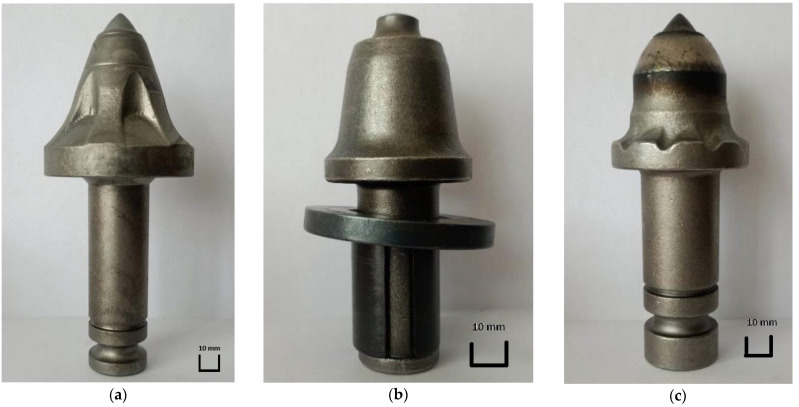
View of the tested milling cutters: (**a**) milling cutter Ø25 mm mounted on bearing balls, (**b**) milling cutter Ø25 mm mounted on expansion sleeve, and (**c**) milling cutter Ø35 mm mounted on bearing balls.

The Ø25 mm ball-bearing mounted milling cutter has an overall length of 142 mm, while its weight is 905 g. It is made of wear-resistant steel with a hardness of about 40 HRC, and the blade itself is made of made of carbide. A view of this milling cutter is shown in Figure 3a. The Ø25 mm sleeve-mounted cutter is an optional accessory that can be installed in the same slot as the ball-bearing-mounted cutter. It is noticeably shorter and lighter than the other components involved in this test. Its overall length is 104 mm, while its weight is 650 g. It is made of wear-resistant steel with a hardness of about 40 HRC, and the blade itself is made of carbide. A view of this cutter is shown in Figure 3b. The Ø25 mm ball-bearing cutter is mounted in a larger socket than the other two cutters. It is the largest and heaviest of the tested cutters. It has a weight of 1295 g and an overall length of 151.5 mm. This means that it is twice as heavy as the Ø25 cutter mounted on an expansion sleeve. Like the other analyzed cutters, it is made of wear-resistant steel with a hardness of about 40 HRC, and the blade is made of carbide. All the cutters tested were made of 42CrMo steel. Meanwhile, the hardness of the test pieces ranged from 44 to 48 HRC. A view of the cutter is shown in Figure 3c. The chemical content of steel used for cutters is presented in Table 1.

**Figure 4:**
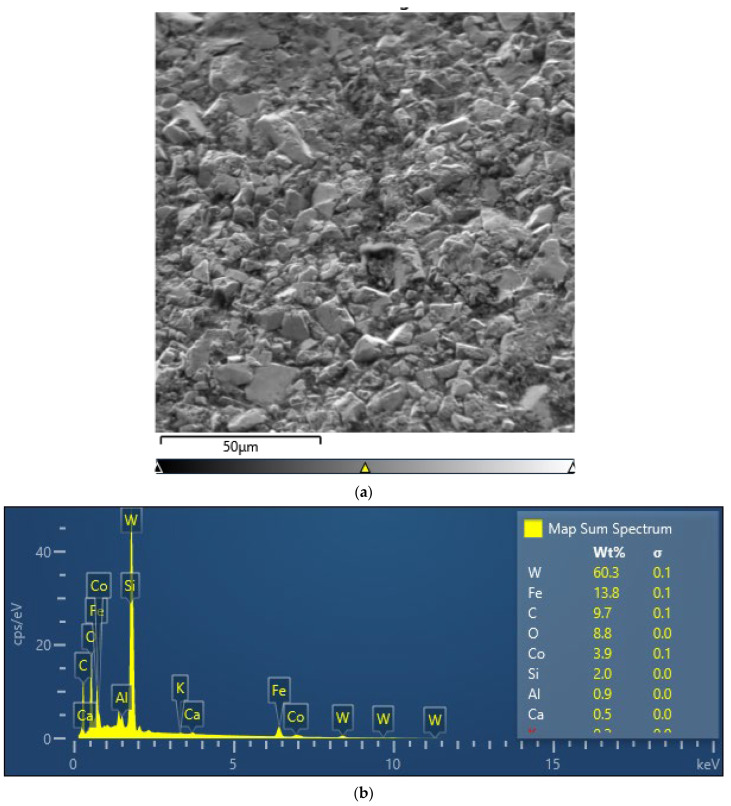
Modified cutter material surface: (**a**) surface view and (**b**) element content.

In addition, field tests where the cutter was most exposed to wear were carried out, in which the producers strengthened the structure of the material by introducing additional elements that increased resistance to abrasive wear (Figure 4).

The field wear test was conducted using Valentini RAMBO 1500 and Valentini IVAN 1500 milling machines and coupled with FENDT agricultural tractors 412 and 716 Vario, respectively.

The field tests were carried out in the Greater Poland region and on a strand of 17.34 km of dirt road. The average width of the road was about 3 m. In order to cover the entire width of the road one machine worked at the right and the other at the left edge of the road. Thanks to the infinitely variable transmission, which allows the speed to be changed smoothly regardless of the engine speed, it was possible to maintain a constant speed at the PTO and thus keep the rotor speed of the machine at the level set by the manufacturer regardless of the operating speed. Due to the characteristics of working on dirt roads, which are often highly irregular in their structure and made of different materials, it is very difficult to conduct a long-distance test at a constant speed. Therefore, it varied between 0.4 and 0.6 km/h during the implementation of field tests.

**Figure 5:**
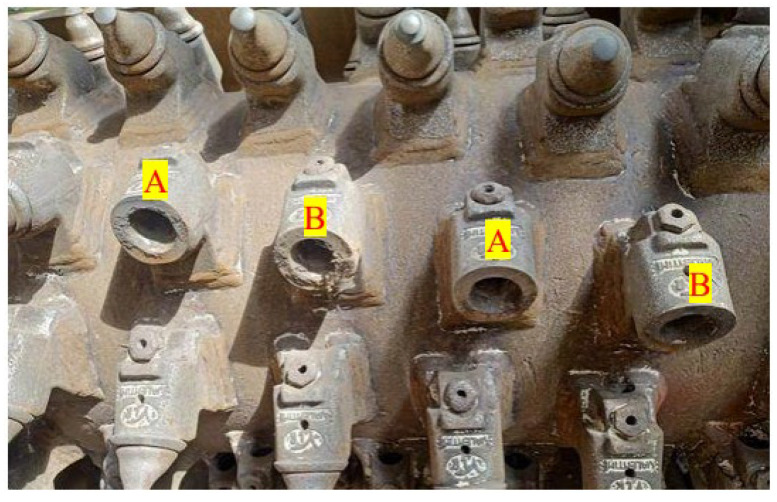
View of the cutter mounting slots: A, B—sample mounting locations.

The tests were performed on 8 samples from each type of cutter. For the Valentini RAMBO 1500 machine, there were 8 samples of the standard Ø25 mm cutter and 8 samples of the Ø25 mm cutter mounted with an adapter sleeve. The samples were arranged in 4 groups each, with every sample being 90° from next cutter. Each row of mounted cutters in the machine had 12 or 13 cutters. Therefore, two groups were arranged from the edges and the other two to the right and left from the center. The Ø35 mm cutters were tested on the Valentini IVAN machine. In this case, elements with a different attachment were not tested. Therefore, two samples were taken in a row from randomly selected rows with each sample being spaced 90° from one another. Elements were taken in such a way that no two working elements from the same position within the width of the machine could be taken. A view of the cutter arrangement is shown in Figure 5.

**Figure 6:**
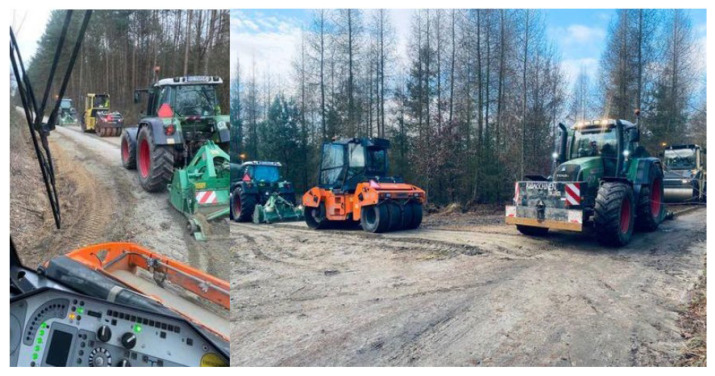
View of dirt road during its renovation process.

The aggregate from which the road was made, as well as that used to fill the largest irregularities, was tested in accordance with accepted standards. It was confirmed that the material meets current standards for use in the construction of dirt roads. Two steel-rubber rollers weighing about 12 tons and a HEN WPF machine (HEN AG, Steinheim an der Murr, Germany) coupled to a FENDT 412 Vario tractor (Fendt, Marktoberdorf, Germany) also took part in the renovation of the selected dirt roads. Below, Figure 6 shows a view taken during the implementation of the renovation of a dirt road.

**Figure 7:**
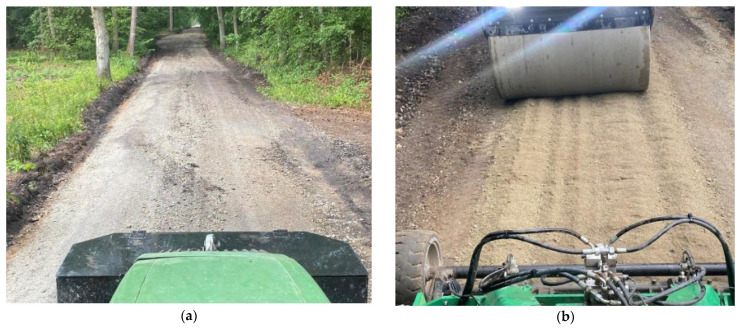
Dirt road during its renovation process: (**a**) road before milling process and (**b**) road after milling process.

Figure 7a shows the road before milling. Figure 7b shows the road after milling process. The mixing of the material and the crushing of the thickest sections after the milling operation can be seen in these figures.

**Figure 8:**
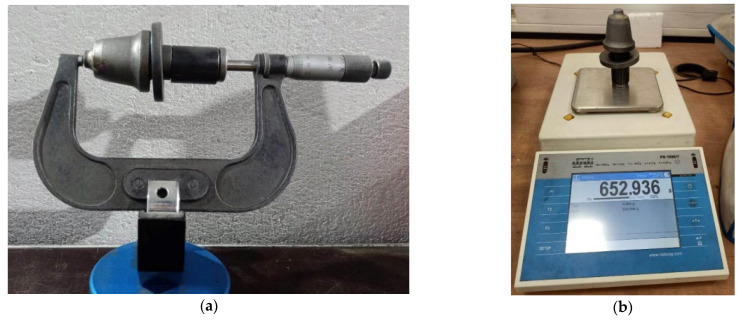
Measurements of sample properties: (**a**) dimensions and (**b**) weight.

The samples subjected to the wear test were numbered, weighed and measured, and then fixed in the milling machines. After working a set distance, the samples were removed from the machines and were cleaned. The final step of this study was to measure the worn samples (measuring geometric properties and weight). The lengths were measured with a VIS micrometer (Vis, Warsaw, Poland) (Figure 8a), the overall length of the cutters was measured, and the weight was checked with a RADWAG PS 1000/Y (Radwag, Radom, Poland) laboratory balance (Figure 8b). The accuracy of the scale and micrometer is 0.001 g and 0.01 mm, respectively.

**Figure 9:**
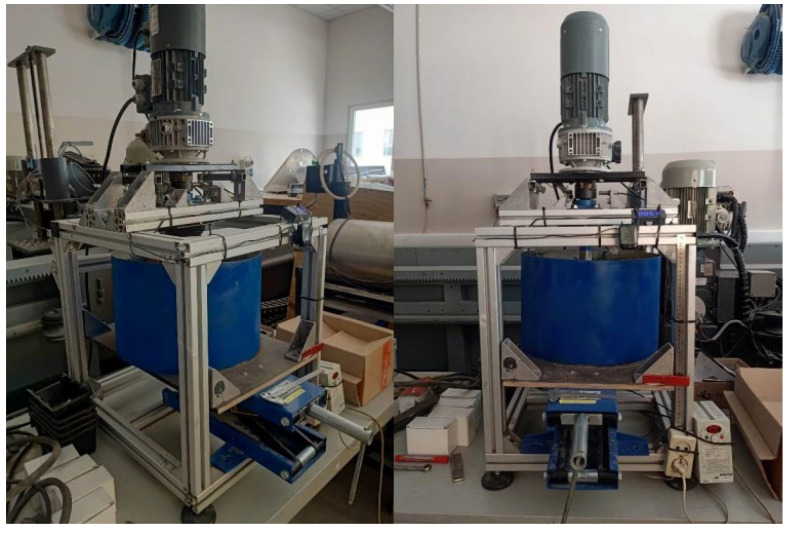
Laboratory stand for testing the wear of samples in the abrasive mass.

**Figure 10:**
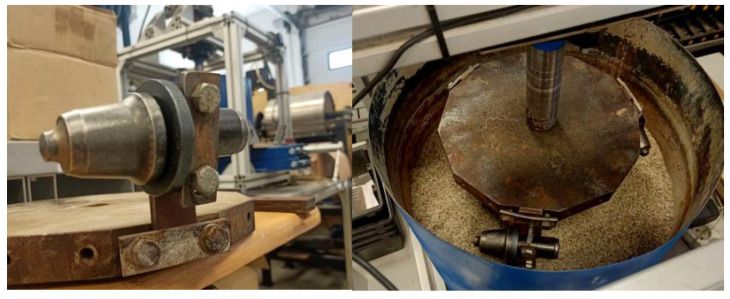
Samples mounted on an abrasive wear test bench.

The second stage of testing was carried out in the laboratory on the dedicated bench, shown in Figure 9. The stand is made up of a movable tank, which contained aggregate in accordance with ASTM G65 [38], and a top section with a rotating head. It was driven by a 0.55 KW geared motor. Before assembly, each sample was numbered and weighed. The samples were fully immersed in the aggregate, and the head speed was 65 revolutions per minute. During the test, 3 samples were mounted on the head at equal intervals in a specific way to prevent their rotation (Figure 10). The bench test lasted 10 h, and was performed in 4 repetitions—2 for each type of sample, totaling 6 samples with 2 types of milling cutters (cutters with a diameter of 25 mm). After 10 h, the samples were disassembled and cleaned in an ultrasonic cleaner. They were then subjected to drying. The cleaned and dried samples were weighed.

## 3. Results and Discussion

After the field research, during which the road was repaired, the road was checked for appropriate quality. The transverse evenness, transverse gradients and degree of compaction were checked. The testing of the evenness and gradients were carried out on a selected straight section of the road with a length of 2307 m. The results show that there are no significant differences between the right and left sides of the road. The lack of significant discrepancies applies to all the studied features of the road. In addition, the results of the test indicate that the road is adequately compacted in accordance with current standards. Table 2 shows the results of cutter wear that occurred during field tests.

**Figure 11:**
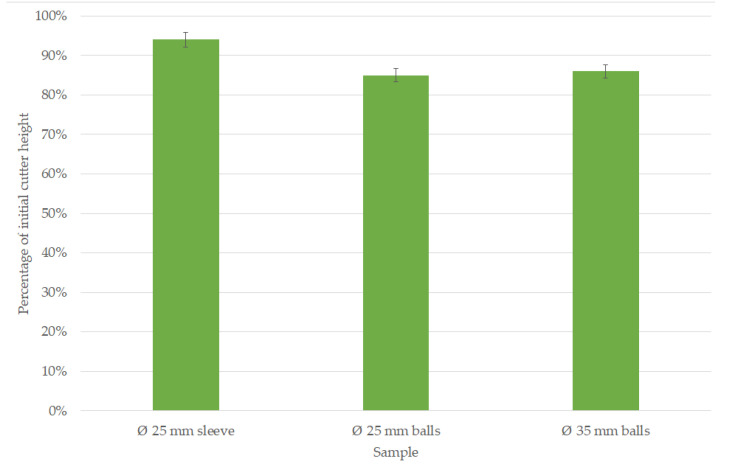
Percentage of the height of a worn component relative to a new component.

Figure 11 presents the percentage of height of the cutter after the milling process of the dirt road in relation to the length of the new tool. The least amount of wear was shown by a Ø25 cutter mounted on an expansion sleeve. Its wear was about 10% less than that of the other cutters. However, these results are reported in relation to the length of the entire tool, including its shank section which varies in length depending on the type of sample analyzed. The other two cutters tested were characterized by a similar length loss and expressed as a percentage.

**Figure 12:**
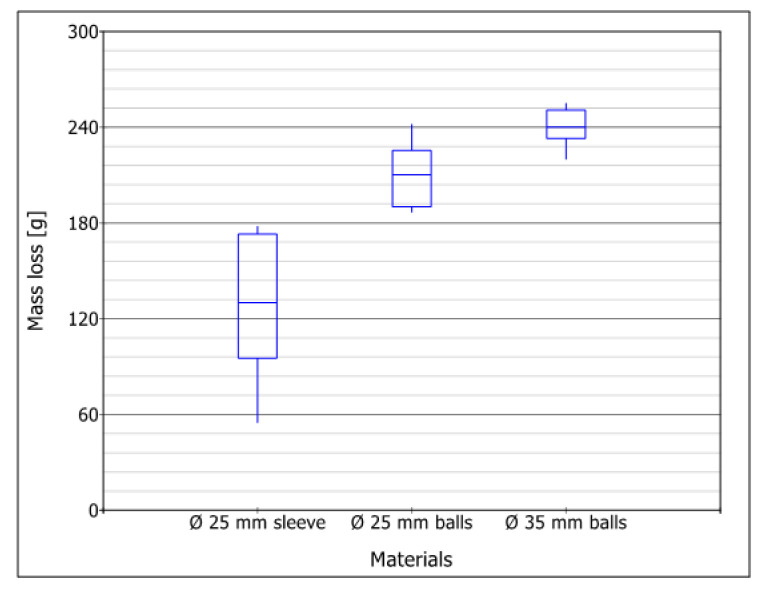
Box plot of the mass loss results for three tested milling cutters based on the field wear test.

The results of the mass loss (wear) of the samples during the implementation of the field tests are shown in Figure 12. Considering these results, it becomes clear that the mass loss increases with the total mass of the cutter. The smallest loss occurred in the Ø25 cutter mounted on an expansion sleeve and was about 130 g, and the largest was for the Ø35 cutter and averaged 240 g. Considering wear as a percentage of the cutter’s original weight, the smallest percentage wear was obtained in the Ø35 cutter (about 19% mass loss) and the largest in the Ø25 ball-mounted cutter (about 23% average mass loss compared to a new cutter).

**Figure 13:**
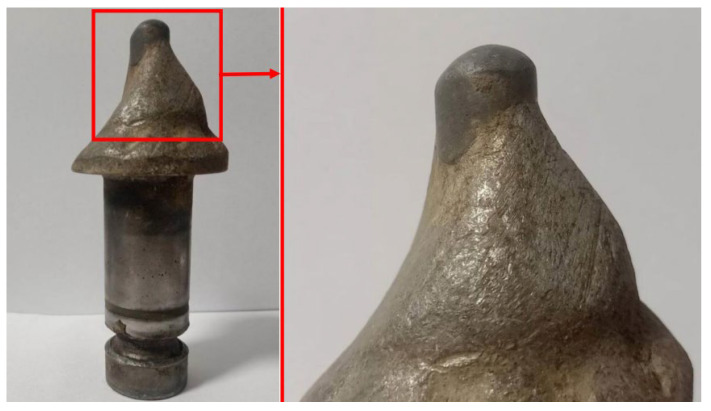
View of a cutter that has undergone accelerated wear.

Considering the results of this research, the visual assessment of the wear of working elements is also important. Cutters mounted in the machine socket should be able to rotate. When rotation becomes difficult or impossible it is followed by accelerated wear. The tool wears out, becomes thinner and more susceptible to breakage or falls out of the embedded carbide, which should bear the main loads during operation. The destruction of the carbide causes the rest of the tool, made of wear-resistant steel, to undergo accelerated wear (Figure 13). In addition, an analysis of the wear results, in millimeters, illustrates the discrepancy between cutter wear depending on the mounting. A clear difference can be seen. Cutters mounted on bearing balls lost about 20 mm from their original length, while cutters mounted with the help of an expansion sleeve lost just more than 5 mm.

**Figure 14:**
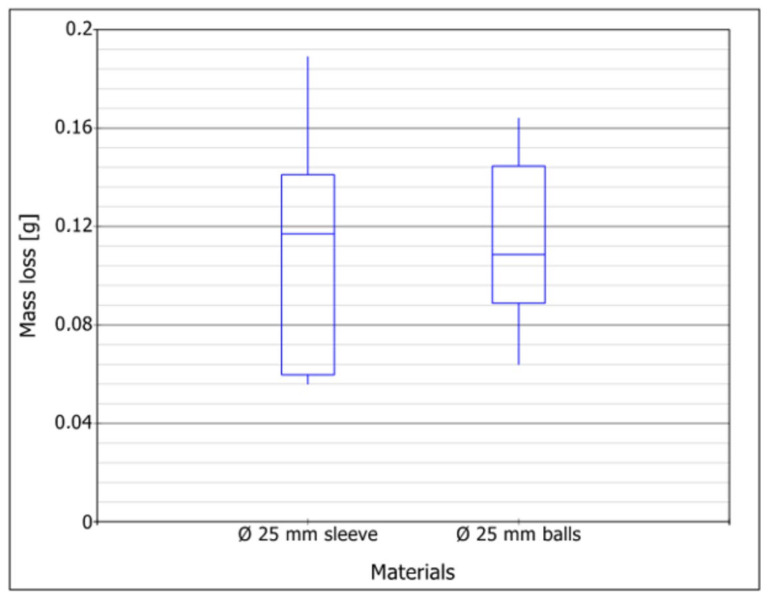
Box plot of the mass loss results for two tested milling cutters based on the laboratory wear test.

The results of the wear test of the samples on the laboratory bench are summarized in Table 3 and Figure 14. The results of laboratory tests confirm the hypothesis that the decisive factor of wear is the ability of the cutter to rotate freely in the socket. Samples mounted with an expansion sleeve, in contrast to the field test, showed higher % wear than those mounted with bearing balls. Accelerated laboratory tests confirmed that it is possible to assess wear on components working in the ground. Cutters mounted with bearing balls tend to wear faster when they stop rotating, and apart from replacement problems, this is their main disadvantage. For milling cutters mounted on an expanding sleeve, no cases of the elements not rotating freely in the seat were observed. This is confirmed by laboratory tests in which samples were immobilized and their wear was comparable.

The results on the wear of cutters during milling of dirt roads indicate important operational aspects of these tools. This study showed that the intensity of use and the type of cutter have a key impact on its service lifespan. In the case of frequent and demanding milling of hard ground, cutters wear faster, requiring more frequent replacement. The wear results obtained are consistent with those available in the literature [6]. Su et al. [30] showed that the shape of the element working in the ground and the material it is made of affect its service lifespan and the reliability of its use. In addition, intense abrasive impact of the sand particles was found on the surface of the worn parts, similar to past studies [2,39]. Statistical tests were added in the analysis of tribological experiment results of the wear test for a better understanding of obtained research results. The fit was again verified using the One-Way ANOVA test. The results of which are presented in Table 4 and Table 5. The calculations indicate that the groups differ significantly; the test statistic (*p*-value) for all contrast cases is less than 0.05.

**Figure 15:**
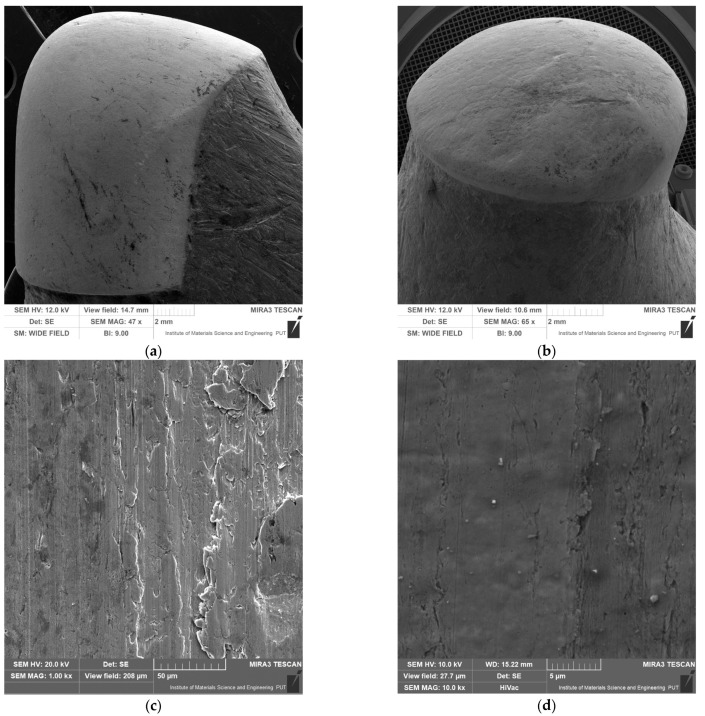
Surface wear analysis: (**a**,**b**) close-ups of manufacturer-reinforced areas that are subject to accelerated abrasive wear and (**c**,**d**) close-ups showing micro-scratching and micro-scouring of the surface of samples after field tests.

Considering Figure 12 and Figure 14, it should be noted that that the analyzed means in each of the tested groups in the field tests are significantly different. Thus, the tribological field wear results are significantly different for all three materials. However, the results obtained from the laboratory tests show that the analyzed cutters in each of the groups tested are similar. The performed test shows that the results of the laboratory wear tests were similar for both materials. The obtained results are most likely due to the fact that constant and equal conditions were maintained for all samples during laboratory tests. For field tests, maintaining identical conditions is not possible. They may be similar for testing each type of cutter, but not identical. Figure 15 shows exemplary traces of wear visible on samples after wear tests. There are traces in the form of micro-scratches and micro-grooves due to the effect of the abrasive. The furrows were arranged along the edges of the milling cutters that stuck into the material of the dirt road.

The obtained test results in the abrasive material are similar to those available in the literature [40,41]. Clear traces of the impact of abrasive particles on the material working in the ground were observed. Wear traces in the form of micro-scratches and micro-grooves are typical for these types of machine elements that are exposed to abrasive wear [42].

## 4. Cost and Efficiency Analysis

In addition to wear tests, monitoring of the time taken to replace worn and damaged cutters, as well as determining the cost of the process, was completed. The tests were performed for two cutters with a diameter of 25 mm (mounted in bearing balls and expansion sleeve). Six replacement trials were carried out and the time required for this operation was recorded during each trial. The results for the six repetitions are similar. The replacement time for cutters mounted on an expanding sleeve is about four times less than for cutters mounted on bearing balls. For a cutter mounted on an expansion sleeve, the replacement time was about 60 s and for a cutter mounted with bearing balls the replacement time was about 240 s. The amount of time required for replacement is a direct result of the complexity of the process. For an expansion sleeve mount, all that is required is to knock out the worn part and hammer in a new one. Both the bushing and the washer that protects the socket from wear remain intact with the cutter even during disassembly, making it unnecessary to remove the bushing cutter and washer separately. The situation is different in the case of the attachment in bearing balls. It is necessary to pay special attention to the necessity of removing all the balls in order to disassemble the cutter. Even if one of them remains, it makes this process effectively impossible. In addition, there is sometimes a situation in which, due to difficult working conditions, the pick stops rotating in the seat. Such a situation affects the accelerated wear of the component. Another problem in such a case is the disassembly of the cutter because the balls are not able to fall out freely when it is not able to rotate freely in the seat and thus makes it impossible to replace the tool. It then becomes much more time-consuming.

In order to identify a working element variant that is more cost-effective to operate, it was necessary to perform a cost analysis. It was assumed that the working element loses 25% of its mass during replacement, and the cost of the labor required to replace the element along with the cost of downtime is PLN 300/h. The data were calculated on the basis of a field survey, and the reference point was the number of kilometers possible to renovate the road without replacing the tool. With this data, and with the use of Formula (1), the cost of milling for the renovation of a kilometer of road was calculated as

(1) $$P=\frac{C_{f}+h\times C_{h}}{d}$$

where

*P*—cost of regeneration of 1 km of road with a given cutter [PLN/km];
*C_f_*—price of the cutter [PLN];
*h*—number of working hours required to replace the worn tool [h];
*C_h_*—cost of an hour of downtime [PLN/h];
*d*—the distance that the replacement cutter is able to work [km].

Also, the calculation includes the cost of purchasing individual cutters, which is as follows:

- Milling cutter Ø25 mm mounted on bearing balls (Ø25 mm balls)—PLN 50;
- Milling cutter Ø25 mm mounted on expansion sleeve (Ø25 mm sleeve)—PLN 40;
- Milling cutter Ø35 mm mounted on bearing balls (Ø35 mm balls)—PLN 160.

**Figure 16:**
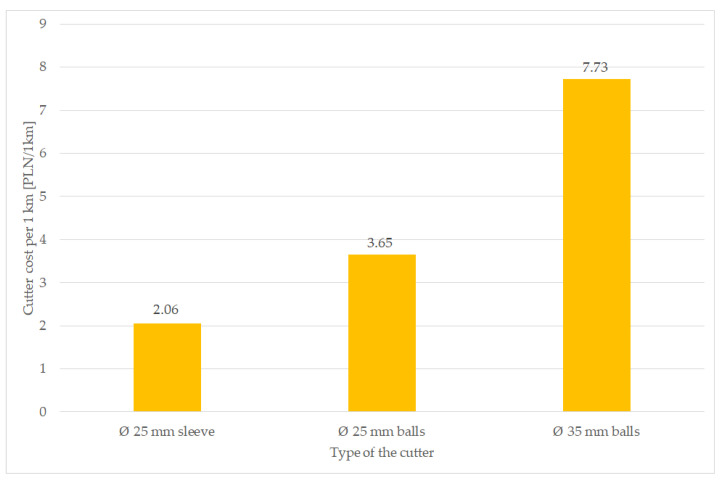
Cutter costs per 1 km of dirt road renovation.

Based on the results of wear, it was determined that the distance after which the cutters should be replaced is every 22 km for a Ø25 cutter mounted on an expansion sleeve, every 19 km for a Ø25 cutter mounted on bearing balls and every 23 km for a Ø35 cutter mounted on balls. On this basis, the cost of the cutter including its replacement per 1 km was calculated (Figure 16), as follows:

- Milling cutter Ø25 mm mounted on bearing balls (Ø25 mm balls)—PLN 3.65;
- Milling cutter Ø25 mm mounted on expansion sleeve (Ø25 mm sleeve)—PLN 2.06;
- Milling cutter Ø35 mm mounted on bearing balls (Ø35 mm balls)—PLN 7.73.

The above costs also include the costs of replacing the cutter, which are as follows:

- Milling cutter Ø25 mm mounted on bearing balls (Ø25 mm balls)—PLN 1.04;
- Milling cutter Ø25 mm mounted on expansion sleeve (Ø25 mm sleeve)—PLN 0.23;
- Milling cutter Ø35 mm mounted on bearing balls (Ø35 mm balls)—PLN 0.86.

The Ø35 milling cutter turned out to be the most expensive, mainly due to its high purchase price compared to the other milling cutters analyzed. If one separates the cost of replacing the tool from its purchase, it turns out that the greatest impact on profitability, in this case, is the tool’s durability and purchase price, rather than the cost of replacing it.

**Figure 17:**
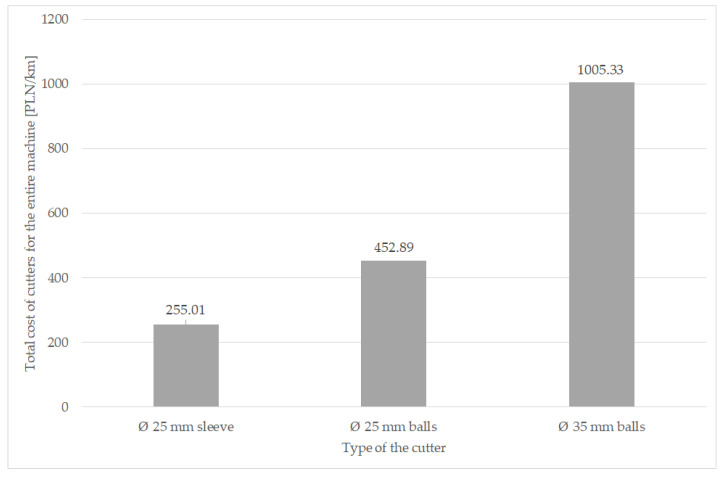
Cutter costs per 1 km of dirt road renovation for one machine.

Considering the number of cutters on the machine, the difference becomes even greater (Figure 17). The Valentini RAMBO 1500 milling machine is equipped with 124 cutters and the IVAN is equipped with 130. When this is factored into the cost, the difference in the cost of the tools needed to renovate the road is several hundred zlotys for each kilometer.

## 5. Conclusions

Based on the completed research, the following conclusions can be made:

- The mass loss and shape-dimensional change depends mainly on the shape of the cutter and the parameters of the abrasive compound; the lowest mass loss was obtained in the Ø25 cutter mounted on an expanding sleeve during testing of the renovation of a dirt road.
- Immobilizing the specimen in one position during the implementation of the laboratory tests resulted in achieving intensive wear in one area of the cutter and smaller wear elsewhere—where the abrasive mass was not pushing on the working element.
- The difference in operating costs for the whole machine between the cheapest and the most expensive variant is about four-fold; therefore, choosing a cutter with a diameter of 25 mm fixed with an expansion sleeve, instead of a cutter with a diameter of 35 mm, saves PLN 750 for each kilometer of dirt road renovated.

Further research should include the possibility of evaluating the effects of soil moisture, different granulometric composition as well as modification of the surface layer on the wear and service lifespan of cutters used for dirt road renovation.

## Figures and Tables

**Table 1:** Chemical content of material used for cutter production.

Content (%)								
C	Mn	Si	P	S	Cr	Ni	Mo	Cu
0.38–0.45	0.50–0.80	0.17–0.37	≤0.035	≤0.035	0.90–1.20	≤0.030	0.15–0.25	≤0.030

**Table 2:** Milling cutter wear test results during field research.

Cutter Type	Ø 25 mm Sleeve				Ø 25 mm Balls				Ø 35 mm Balls			
	Dimension mm		Weight g		Dimension mm		Weight g		Dimension mm		Weight g	
	Before	After	Before	After	Before	After	Before	After	Before	After	Before	After
1	104.38	97.52	648.945	480.616	142.33	118.59	905.345	688.681	151.2	125.01	1293	1053
2	104.29	102.01	649.234	555.338	142.1	134.67	906.04	706.002	151.492	123.53	1293	1053
3	104.67	96.14	650.245	475.594	142.67	124.82	906.569	702.927	151.401	125.13	1294	1044
4	104.58	100.34	650.693	551.1	142.79	116.63	906.221	681.556	151.594	139.31	1293	1042
5	104.44	103.55	650.329	595.32	142.42	115.15	905.893	680.176	151.603	140.63	1295	1056
6	104.31	100.63	649.998	542.317	142.6	122.06	905.934	664.084	151.382	140.33	1293	1062
7	104.61	97.69	650.801	498.39	142.25	116.69	905.873	719.025	151.499	125.8	1294	1074
8	104.87	86.30	648.988	471.14	142.11	116.03	906.29	766.304	151.508	125.83	1295	1040
Standard Deviation	0.187	4.999	0.702	45.655	0.241	6.160	0.338	31.379	0.123	7.314	0.829	11.486

**Table 3:** Milling cutter wear test results during laboratory research.

Cutter Type	Ø 25 mm Sleeve		Ø 25 mm Balls	
	Weight g		Weight g	
	Before	After	Before	After
1	650.414	650.353	905.339	905.242
2	648.915	648.859	906.978	906.814
3	649.672	649.547	905.446	905.382
4	647.068	646.947	905.684	905.577
5	652.994	652.881	906.024	905.886
6	648.271	648.082	905.981	905.871
Standard Deviation	1.863	0.049	0.045	0.034

**Table 4:** Mean comparison for field wear test.

Contrast	Mean Difference	Pooled Standard Error	*p* Value
Ø 25 mm sleeve–Ø 25 mm balls	−76.249	16.3323	0.000132
Ø 25 mm sleeve–Ø 35 mm balls	−112.07275	16.3323	8.785396 × 10^−7^
Ø 25 mm balls–Ø 35 mm balls	−35.82375	16.3323	0.039672

**Table 5:** Mean comparison for laboratory wear test.

Contrast	Mean Difference	Pooled Standard Error	*p* Value
Ø 25 mm sleeve–Ø 25 mm balls	−0.0025	0.024383	0.920363

## Data Availability

The data presented in this study are available on request from the corresponding author.
